# Experimental investigation and AI-based prediction of engine performance and emissions using CeO_2_-enhanced biodiesel blends

**DOI:** 10.1038/s41598-025-28932-4

**Published:** 2025-12-13

**Authors:** Ganesh K, D K Ramesha, Kapilan Natesan, Norkhairunnisa Mazlan, Sadashiva Prabhu S

**Affiliations:** 1 NITTE (Deemed to be University), Nitte Meenakshi Institute of Technology, Yelahanka, Bengaluru, Karnataka 560064 India; 2 Department of Mechanical Engineering, University of Visvesvaraya College of Engineering, K R Circle, Bengaluru, Karnataka 560001 India; 3 Department of Aerospace Engineering, Faculty of Engineering, Universiti Putra Malaysia (UPM), 43400 Serdang, Selangor Malaysia; 4 Department of Mechanical and Industrial Engineering, Manipal Institute of Technology, Manipal Academy of Higher Education, Manipal, Karnataka 576104 India

**Keywords:** Biodiesel, Nanoparticles, Engine tests, Prediction, Random forest regressor model, Energy science and technology, Engineering, Environmental sciences

## Abstract

The global depletion of fossil fuel resources and growing environmental concerns have driven the pursuit of sustainable alternative energy sources, with biodiesel emerging as a promising option. In the present work, experimental and AI prediction studies examine the combustion, performance, and emissions of a B20-blended fuel engine. The biodiesel was derived from used temple oil, and CeO_2_ nanoparticles (NPs) were used as additives. The experiments conducted at a constant engine speed with various loads reveal that CeO_2_ NPs at a 100-ppm concentration significantly improve brake thermal efficiency (BTE). The specific fuel consumption decreases with increasing CeO_2_ NPs concentration, indicating enhanced fuel efficiency. The higher cylinder pressure and net heat release (NHR) further highlight the improved combustion characteristics. The emission analysis reveals a notable decline in hydrocarbons, carbon monoxide, and nitrogen oxides levels, with CeO_2_ NPs at a 100-ppm concentration achieving the lowest emissions due to superior fuel atomization, improved combustion efficiency, and enhanced thermal management facilitated by the presence of CeO_2_ NPs. The B20 blend with CeO_2_ NPs at a 100-ppm concentration demonstrated superior performance metrics at maximum load, as evidenced by a 1.57% increase in BTE, a 2.83% rise in cylinder pressure, and a 5.95% increase in NHR compared to conventional diesel. A significant reduction in pollutant emissions, such as carbon monoxide, decreased by 66.67%, hydrocarbons by 13.51%, and nitrogen oxides by 6.04% relative to the B20 blend. The validation of experimental data using a random forest regressor model demonstrates strong predictive accuracy, characterized by low mean squared errors and high R^2^ scores across various performance metrics. Overall, the findings of this work confirm that the B20 blend with CeO_2_ NPs is a sustainable and efficient replacement for traditional diesel fuel.

## Introduction

The worldwide requirement for energy resources is increasing rapidly, with forecasts predicting almost 60% rise by 2035. This rise in energy usage has resulted in the depletion of fossil fuel supplies^1^. Additionally, the growing fuel consumption per person has led to higher fuel prices. At the same time, the widespread reliance on fossil fuels is widely acknowledged as a major factor in environmental pollution, as the burning of these fuels emits harmful gases^2^. Notably, the transport sector’s greenhouse gas (GHG) emissions are rising faster than any other sector, with 96.3% of its fuels in 2018 derived from fossil sources. It contributes 15% of global GHG emissions and 23% of energy-related CO_2_ emissions^3^. Therefore, the need of the era in combating climate change is the development of non-polluting energy sources. Furthermore, petroleum-importing countries face energy and foreign exchange crises, highlighting the need for locally sourced alternative fuels and options like alcohol, biodiesel, and vegetable oils that support sustainable development and environmental protection^4^. Alternative fuels, such as green hydrogen and biofuels, are seen as key to reducing vehicle emissions. However, concerns about their sustainability, risks, and end-of-life impact require thorough evaluation^5^. Biodiesel contains zero sulfur and fewer aromatic compounds, along with oxygen-rich ingredients, resulting in lower emissions of CO_2_, HC, and particulate matter. Furthermore, its higher cetane number enhances ignition characteristics in engines compared to traditional diesel fuel^6^. As evidenced, biofuels like biomethanol and biodiesel can cut shipping industry emissions by 25% to 100%, but the current supply meets only 15% of demand. Besides, the challenges such as oxidation, ecological impact, feedstock limits, and economic and technical barriers must be resolved for large-scale adoption^7^.

Having stated biofuels can reduce fossil fuel dependence and emissions and aid the global energy transition, they may cause fuel injection problems, viscosity issues, and involve sustainability concerns^8^. Hence, introducing fuel additives of nano-scale dimensions has been a crucial domain of investigation to resolve the above issues in biodiesel-powered diesel engines. The emerging research indicates that the incorporation of nano-additives into diesel engine fuels can diminish auto-ignition temperature, evaporation time, and ignition delay. Further, it improves the atomization rate and mitigates incomplete fuel combustion^9^. Some of the beneficial effects of additives to biodiesel, examined by a few researchers, are discussed in the following sections.

Incorporating molybdenum disulfide with a mixture of ethanol, butanol, and acetone into castor biodiesel resulted in the highest decrease in emissions of CO, CO_2_, and NO_x_, which were reduced by 38.8%, 36.3%, and 43.09% respectively, compared to diesel^10^. For the same castor biodiesel blend, B20, added with 100 ppm BaTiO_3_, demonstrated encouraging outcomes, showing a 6.47% increase in brake thermal efficiency (BTE) and 8.1% decrease in Brake specific fuel consumption (BSFC) compared to diesel^11^. Another biodiesel, Mauha biodiesel, showed BTE improvements of 1.58%, 1.62%, and 2.34% with the inclusion of 40, 80, and 120 ppm of Fe_2_O_3_ nanoparticles (NPs), respectively^12^. A study investigated the influence of liquid and metal oxide additives on waste cooking oil (WCO) biodiesel blends, specifically B30 and B40 blends, using diethyl ether (DEE) and NPs as additional components. The findings indicated that the BTE of B30 with NPs reached 29.57%, which is similar to the efficiency of neat B20 operation at 29.72%. The most significant reductions in HC and smoke emissions were recorded for B40 with NPs, demonstrating a decrease of 16% and 11.7%, respectively, compared to neat B20 fuel operation. The B30 combined with DEE and NPs exhibited the lowest NO_x_ emissions, which were 6% lower than those of the B20 blend^13^. Another study shows that the B20 blended with ethanol (5%) and supplemented with Al_2_O_3_ NPs, significantly boosts combustion efficiency and lowers CO, HC, and smoke emissions^14^. A few researchers also studied the combined effect of dual NPs. The addition of dual NPs such as TiO_2_ and Al_2_O_3_ to the biodiesel improves BTE and reduces emissions^15^. However, the stability of NPs in the biodiesel is a challenge and it should be studied in detail^16^. The NPs of CeO_2_ exhibit some unique characteristics in the combustion of biodiesel compared to other NPs and require detailed discussion.

### Choice of CeO_2_ and suitability of the same for engine fuel applications

**Fig. 1:**
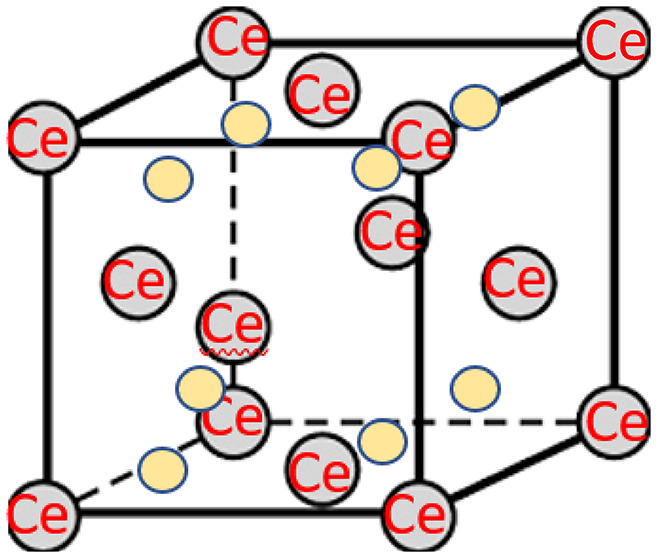
Structure of CeO_2_.

A rare earth element known as cerium exists naturally with two oxidation states, + 3 and + 4. In its + 4-oxidation state, ceria acts as a strong oxidizing agent and is classified as a non-trivalent rare-earth ion. The + 3 state of cerium closely resembles other trivalent rare earth elements. The ceria is a stable, non-toxic refractory substance that has a melting point of 2600 °C and a density of 7.13 g/cm³. It features a fluorite crystal structure, which includes eight-coordinate Ce^4+^ ions and four-coordinate O^2−^ ions. Figure 1 illustrates the face-centered cubic (FCC) structure of stoichiometric CeO_2_, with large red spheres representing the four-coordinate oxygen anions and small blue spheres depicting the eight-coordinate cerium cations^17^.The CeO_2_ NPs can be chosen as the nano-additive due to their high oxygen storage capacity (500 µmol/g), scavenging of free radicals, and good redox properties, which supply extra oxygen during the combustion process and facilitate complete fuel oxidation. Their size, good morphology and good catalytic characteristics can make them highly effective in the combustion process due to the presence of a large reactive surface area. These characteristics can render CeO_2_ very effective in enhancing engine performance and decreasing emissions in comparison to other NPs. At elevated temperatures, the CeO_2_ releases oxygen to create an oxygen-deficient variant that preserves the fluorite structure. The CeO_2_ NPs find extensive applications in catalysis, polishing, optical systems, mixed conduction, fuel cells etc^18^.

A limited number of researchers have investigated the engine performance using CeO_2_ NPs. It is revealed that the thermal performance characteristic curves of a lemongrass- CeO_2_ nano-emulsion fuel result in 17.21% improvement in BTE compared to mineral diesel fuel^19^. Kapoor et al.^20^ showed that adding DEE to the biodiesel added with the same NPs improves performance characteristics and lowers harmful emissions. Another study suggests that incorporating NPs into biodiesel can reduce engine emissions, potentially lowering exposure to carcinogens and eye irritants that trigger allergic responses in humans and animals^21^. Furthermore, the effect of the sizes of NPs also has a detrimental effect on performance and emission, and it is reported that the NPs of size 50 nm achieved the best performance metrics and minimized exhaust emissions across all compression ratios^22^. However, another study demonstrates that NPs with a size of 30 nm achieved the best performance and the lowest engine emissions, highlighting the importance of optimizing NP dosage when added to biodiesel^23^. As a relatively new method, few authors have studied the use of dual NPs, such as Al_2_O_3_ and CeO_2_ NPs, to obtain biodiesel reductions in HC, NO_x_, and CO emissions by 30.73%, 1.27%, and 44.13%, respectively, with an increase in BTE^24^. Another study with a dual NPs mixture showed reductions in the emissions of NO_x_, CO, HC, and smoke opacity of 24.67%, 33.33%, 23.08%, and 34.01%, respectively, compared to pure biodiesel^25^.

Many studies cited above consider conventional biodiesels derived from edible oil sources. It is advisable to utilize biodiesels available in lesser quantities from unutilized wastes, such as dairy waste and temple oil, which are available in larger quantities in India. It is also observed that no extensive studies have been conducted on biodiesels derived from these sources. In this context, the used temple oil (UTO) was utilized as a raw material for biodiesel production and subsequently used as fuel in the engine. Furthermore, having been aware of the benefits of CeO_2_, it is considered a beneficial additive to this biodiesel.

The novelty of this work includes the use of temple oil, a non-edible and underutilized waste resource, as a sustainable feedstock for biodiesel production, which offers both environmental and socio-economic benefits. The present study uniquely investigates the influence of CeO_2_ NPs addition on the physicochemical characteristics of UTO methyl ester (UTOME), specifically examining parameters such as heating value, cetane number, and kinematic viscosity. This exploration is particularly significant since biodiesel typically exhibits lower volatility and a higher flash point, and the incorporation of NPs offers a novel approach to overcoming these inherent limitations and enhancing fuel performance. From the literature, NP concentrations of 50, 75, and 100 ppm have commonly been studied; therefore, in this work, CeO_2_ NP concentrations of 50, 75, and 100 ppm were selected, aligning with the typical dosage ranges reported by other researchers. These concentrations are deemed appropriate for optimizing combustion and emission characteristics while avoiding NPs agglomeration or operational problems. The concentrations lower than 50 ppm tend to yield negligible performance gains, whereas concentrations exceeding 100 ppm can lead to inadequate dispersion and injector fouling. The selected range thus provides a compromise between fuel stability and improved combustion behavior. The initial dispersion experiments verified a consistent distribution of NPs in the blends at these concentrations, which validates the appropriateness of this range for engine testing in the present investigation.

### Application of data processing and machine learning

In recent years, various Artificial Intelligence (AI) based optimization techniques have been used to predict the performance of the IC engine with various fuel blends, to reduce the need for extensive physical testing^26^. It is suggested that the AI-based analysis of a variable compression ratio engine using biodiesel blends, combined with uncertainty modeling, enhances the accuracy and reliability of performance and emission predictions^27^. The application of Machine Learning (ML) methodologies has also been shown to be effective in forecasting the behaviour of complex systems, encompassing engines^28^. The ML is applied in various biodiesel-related processes, including optimizing production, predicting fuel properties, and enhancing process control through data-driven analysis and modeling^29^. A study reported that the Artificial Neural Network (ANN) model yields higher prediction accuracy for emissions, outperforming regression in some cases^30^. Another study explored alternative fuels in IC engines using ANN and RSM (Response Surface Methodology), to predict engine performance and the experimental data based on load, speed, compression ratio, and biodiesel blends were used for training. The results indicate both methods are effective for accurate prediction and optimization of engine parameters with minimal error^31^. Furthermore, the utilization of ML in reactivity-controlled CI engine operations fuelled by CNG and algal biodiesel presents a promising strategy for maintaining efficient diesel engine performance^32^.

With the introduction of NP utilization in engines, the ML algorithms can be employed to gain deeper insights into the complex interactions between NPs and overall engine performance^33^. A comparative analysis among the ML algorithms is conducted, employing performance evaluation metrics to comprehensively assess their predictive capabilities^34^. Incorporating the ML techniques into this particular engine application offers significant potential to enhance the sustainability and efficiency of diesel engines while reducing their environmental impact^35^. Despite extensive research on predicting engine performance, combustion, and emissions, current ML models often struggle to accurately capture the complex, nonlinear relationships in biodiesel–nano additives datasets. It is suggested that advanced ML models could significantly improve the prediction accuracy of performance, emissions, and calibration efficiency in rotary engines^36^. A further improvised predictive method, the Random Forest Algorithm (RFA), is explained below.

**Fig. 2:**
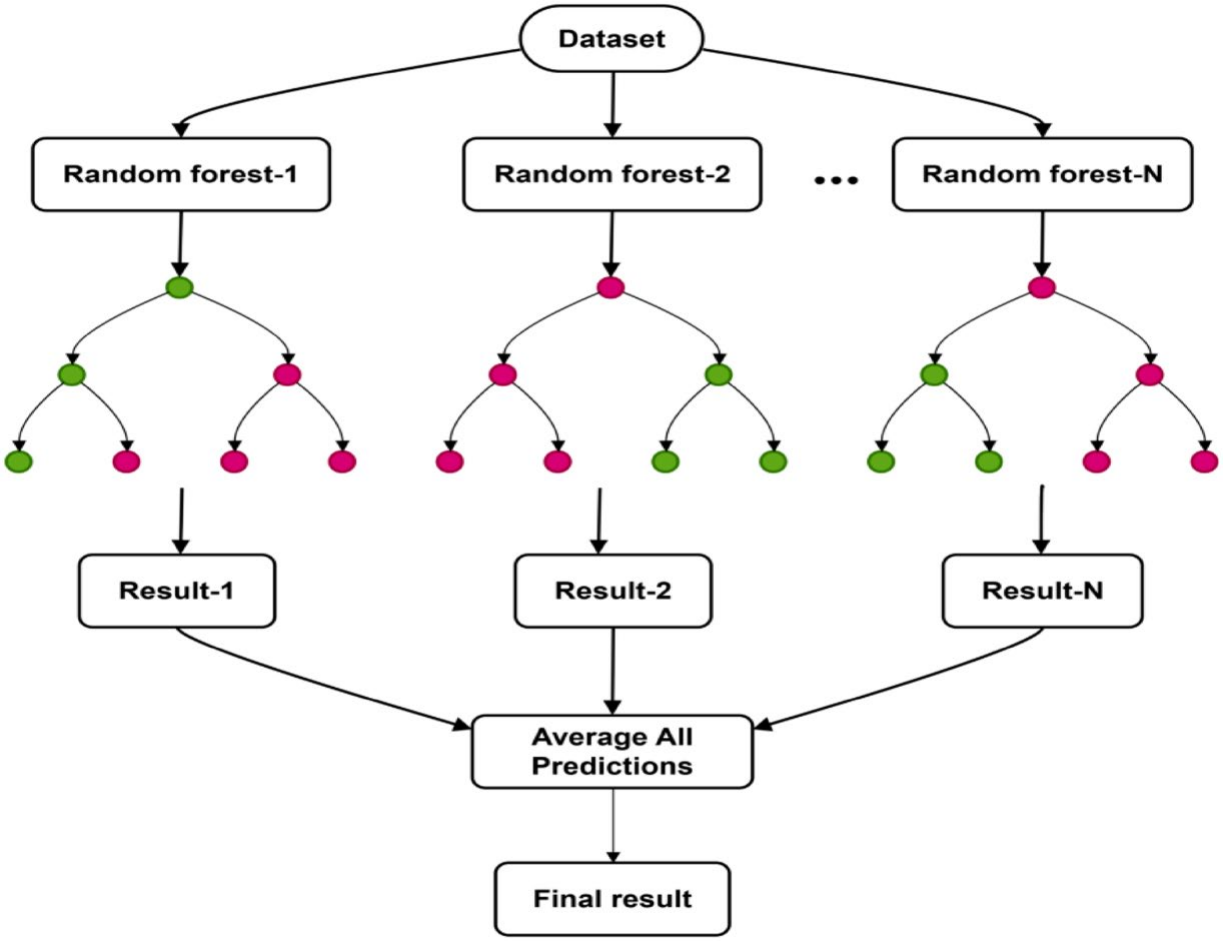
Working principle of the Random Forest Algorithm^37^.

Figure 2 is a representation of how the RFA works. As observed, the test sample is evaluated by several decision trees, each trained on a different data subset. Each tree makes a prediction, and these predictions are averaged or combined to produce a final, overall result. This process improves the model’s accuracy and reduces overfitting^34^. Apart from the above, the RFA addresses this limitation by integrating multiple decision trees, which allows for robust predictions even with small, noisy, or highly variable experimental data. Its capacity to manage feature interactions, reduce overfitting, and perform internal validation makes it a highly effective tool for reliable and precise prediction of engine performance, combustion, and emission characteristics when compared to other ML methods. In that context, the RFA, as discussed above, is applied to do optimization in this work. This combined biodiesel blending, nano additive enhancement, and AI-assisted analysis demonstrates a novel and holistic advancement in clean and efficient engine fuel research.

## Materials and methodology

### Preparation of biodiesel

**Fig. 3:**
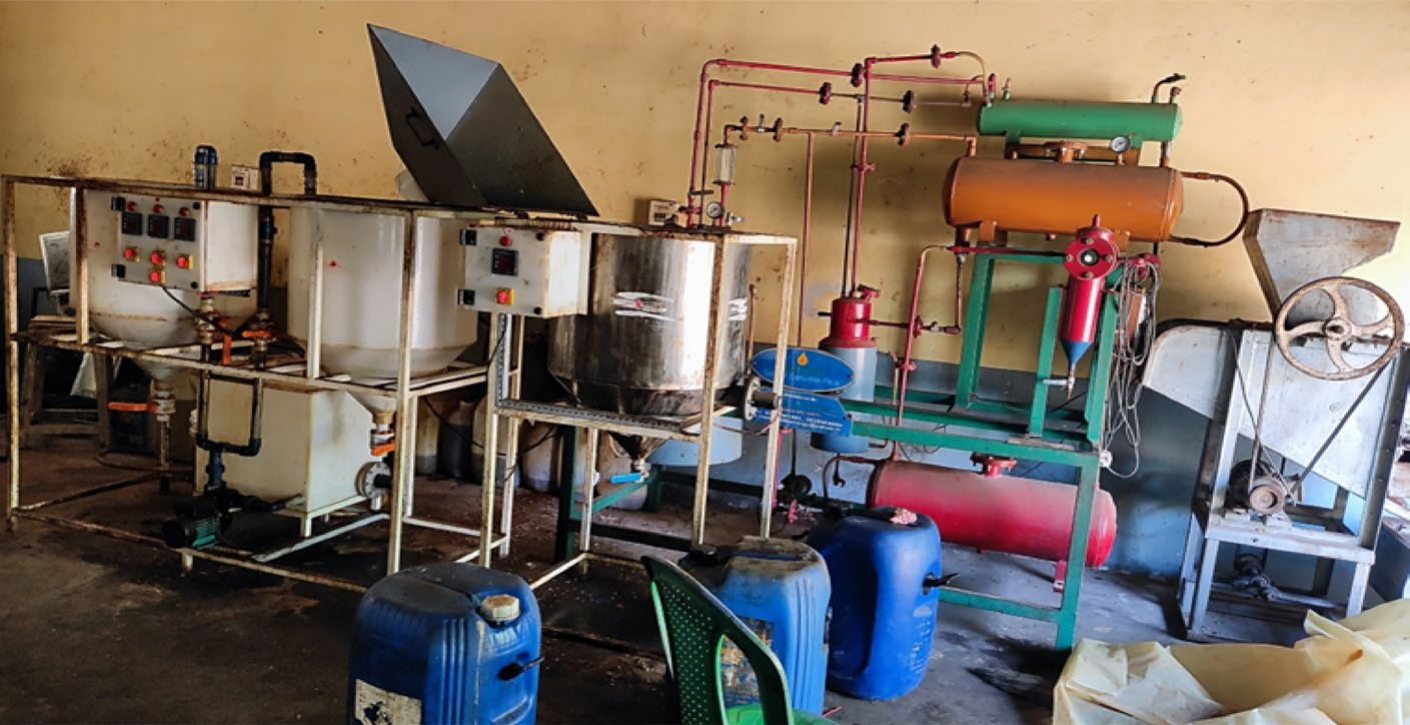
Experimental setup of the Transesterification process.

**Fig. 4:**
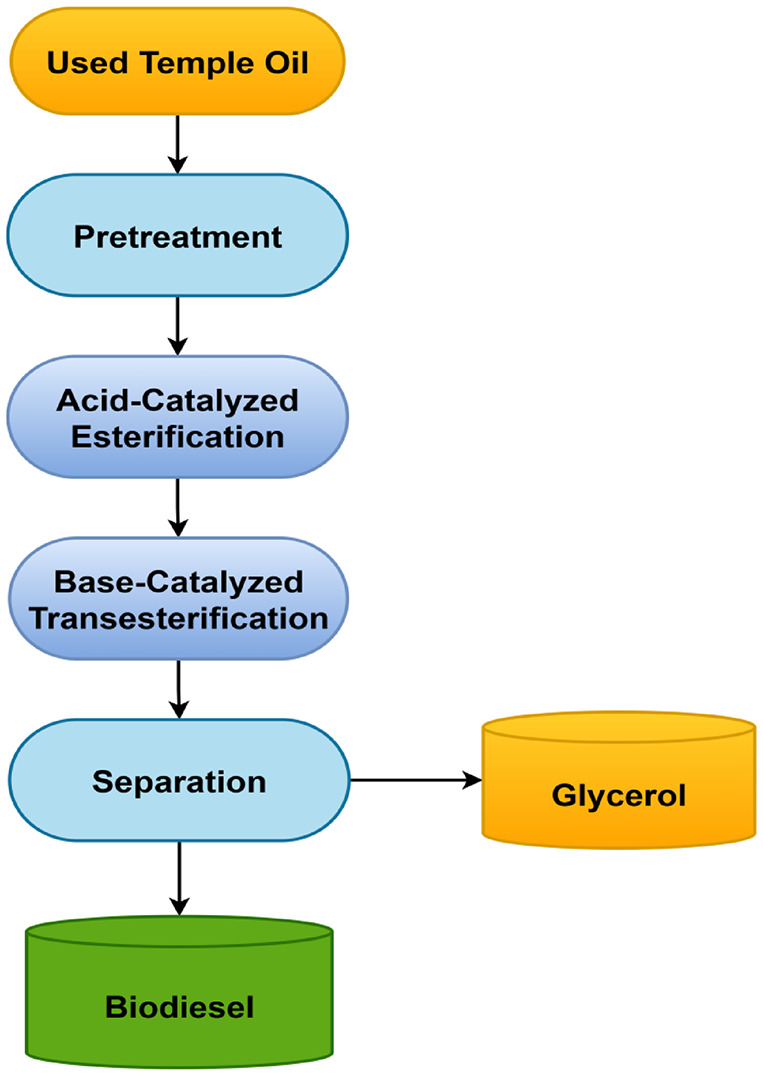
Schematic diagram of biodiesel production from Used Temple Oil.

The UTO was collected from famous temples in Karnataka state, India and it was filtered to remove impurities and Fig. 3 shows the experimental setup used for UTOME. The preparation of biodiesel from UTO involves a process called transesterification (two-step), using methanol and sulfuric acid in the first step to reduce acid value below 1%, and methanol and potassium hydroxide in the second step to produce biodiesel and glycerol as a byproduct. The transesterification process is essential because it reduces the oil’s viscosity, making it more appropriate for application in diesel engines without the need for engine modifications. The raw UTOME was subjected to water wash using warm water to remove excess catalyst and other substances which was produced during the reaction. The reaction temperature and reaction time considered in this work were 60 °C and 90 min. The oil-to-methanol ratio considered in this work was 1:6, and Fig. 4 depicts the schematic of the procedure followed to produce UTOME.

### Fuels used

The fuel used in this work is listed below, along with its abbreviations.

1. Diesel.
2. B20UTOME (B20 Used Temple oil Methyl Ester).
3. B20UTOME50CeO_2_ (B20UTOME + 50 ppm CeO_2_ NPs).
4. B20UTOME75CeO_2_ (B20UTOME + 75 ppm CeO_2_ NPs).
5. B20UTOME100CeO_2_ (B20UTOME + 100 ppm CeO_2_ NPs).

In this investigation, five distinct fuel combinations are examined. These include conventional Diesel and a B20UTOME biodiesel blend, comprising 20% UTOME and 80% diesel. So, the B20UTOME blend is fortified with varying concentrations of cerium oxide nano-additives at 50, 75 and 100 ppm. The CeO_2_ NPs in the B20UTOME blends are anticipated to optimize combustion efficiency and minimize emissions, rendering these biodiesel blends more sustainable and efficient alternatives to conventional diesel.

### Preparation of fuel blends

**Fig. 5:**
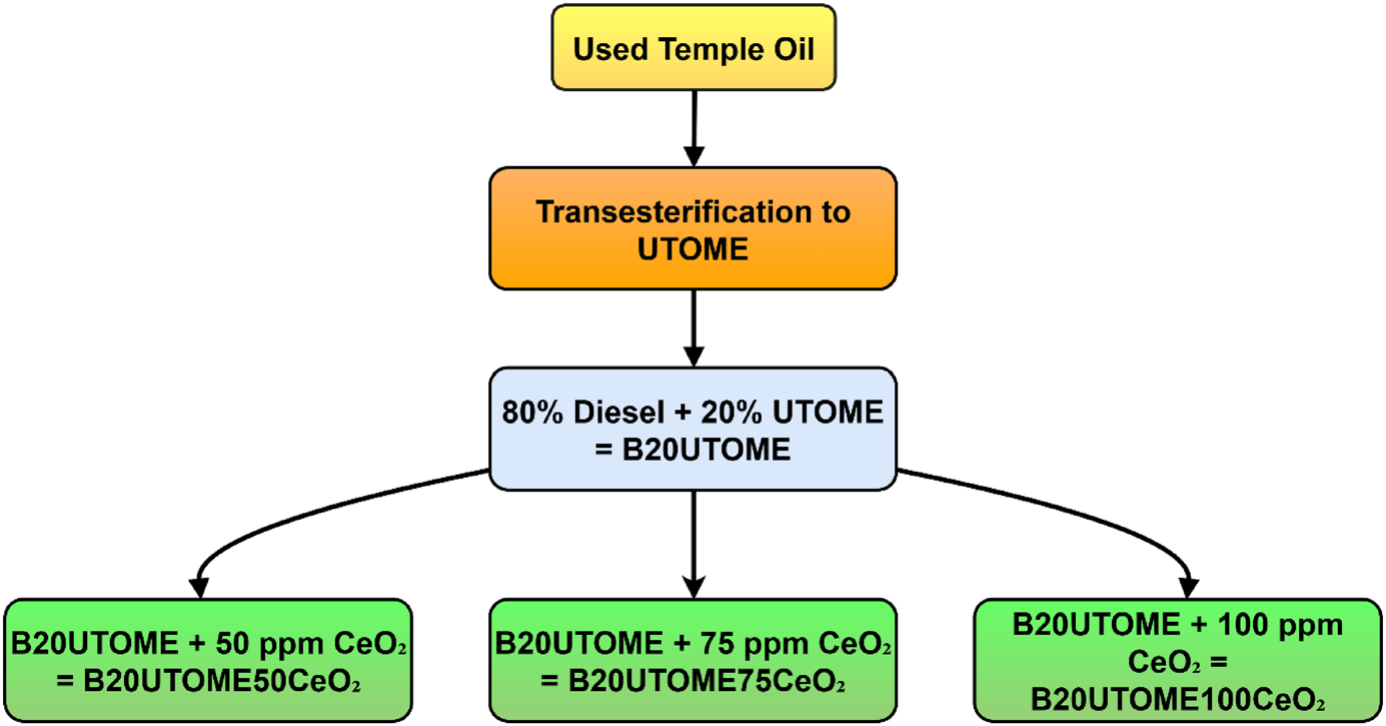
Flow diagram illustrating the preparation of B20 biodiesel blends from UTO with varying concentrations of CeO_2_ NPs.

In this study, UTO was transformed through a transesterification process to produce UTOME, which was then blended with conventional diesel in an 80:20 ratio to form B20UTOME. The sorbitan monooleate (Span 80) was used as a surfactant. To further enhance the fuel properties and reduce emissions, CeO_2_ NPs were incorporated into the B20UTOME blend at varying concentrations of 50, 75 and 100 ppm, resulting in B20UTOME50CeO_2_, B20UTOME75CeO_2_, and B20UTOME100CeO_2_, respectively. The above combinations are detailed in Fig. 5. These NPs enhanced fuel blends were prepared to evaluate the catalytic impact of CeO_2_ on combustion performance and emission reductions, leveraging the improved atomization and oxygen content of the NPs. The different concentrations were selected to investigate the influence of CeO_2_ NPs on emissions and performance metrics, providing insights into the potential of B20UTOME with CeO_2_ as a cleaner alternative fuel.

### Properties of Fuels: Diesel, UTO, UTOME & B20UTOME

The properties of fuel are listed in Table 1.

**Table 1:** Properties of Fuels - Diesel, UTO, UTOME, B20UTOME, B20UTOME50CeO_2_, B20UTOME75CeO_2_, and B20UTOME100CeO_2_.

Sl. No	Property	Test type	Diesel	UTO	UTOME	B20 UTOME	B20 UTOME 50CeO_2_	B20 UTOME75 CeO_2_	B20 UTOME100 CeO_2_
1	Density (kg/m³)	D4052	850	912	868	860	861	862	863
2	Kinematic viscosity @ 40 °C (mm^2^/s)	D 445	2.50	25.80	4.30	3.60	3.62	3.64	3.66
3	Iodine value (mg I_2_/100 g)	D5768	–	130	119	105	105	105	105
4	Acid number (mg KOH/g)	D 664	0.30	3.60	0.75	0.55	0.54	0.54	0.53
5	Higher heating Value (MJ/kg)	D4809	43.00	38.50	39.20	40.00	40.15	40.25	40.30
6	Cetane number	D 613	46	45	50	48	48.5	49	49.5
7	Flash point (°C)	D 93	56	165	110	75	74	72	71

The physicochemical properties of B20UTOME closely resemble those of conventional diesel fuel, positioning it as a viable alternative. It exhibits analogous density and kinematic viscosity characteristics, facilitating unimpeded flow and combustion within diesel engine systems. Furthermore, its elevated heating value and cetane number are adequate to ensure efficient energy generation and ignition performance, rendering B20UTOME a sustainable substitute for diesel.

### Properties of CeO_2_ NPs

The properties of CeO_2_ NPs are listed in Table 2. Some of the properties are helpful in understanding how CeO_2_ NPs are helpful in understanding performance and emission characteristics.

**Table 2:** Properties of CeO_2_ (Cerium Oxide), Al_2_O_3_ (Aluminium Oxide) NPs.

Property	Al_2_O_3_	CeO_2_
Colour	White	Yellowish or pale yellow
Morphology	Spherical or Irregular	Spherical or Cubic
Average particle size (APS)	30 nm	20 nm
Specific surface area (SSA)	100 m^2^/g	80 m^2^/g
Oxygen storage capacity (OSC)	5 µmol/g	500 µmol/g
Thermal conductivity	~ 30 W/m·K	~ 12 W/m K
Catalytic activity	Low (5 µmol/g OSC)	High (500 µmol/g OSC)

The CeO_2_ NPs exhibit a pale-yellow color and a spherical or cubic morphology, with an average particle size of 20 nm. This material possesses a high oxygen storage capacity and considerable catalytic activity, rendering it highly effective in improving combustion and reducing emissions. Furthermore, CeO_2_ NPs demonstrate a thermal conductivity of approximately 12 watts per meter-Kelvin, making them suitable for applications in fuel additives. Ultrasonication was used to achieve a uniform distribution of CeO_2_ NPs throughout the fuel mixtures. The preparation process included an initial 10-minute period of magnetic stirring, followed by 20 min of ultrasonication to ensure a stable and consistent dispersion of the NPs in the B20UTOME fuel blend.

### Experimental procedure

**Fig. 6:**
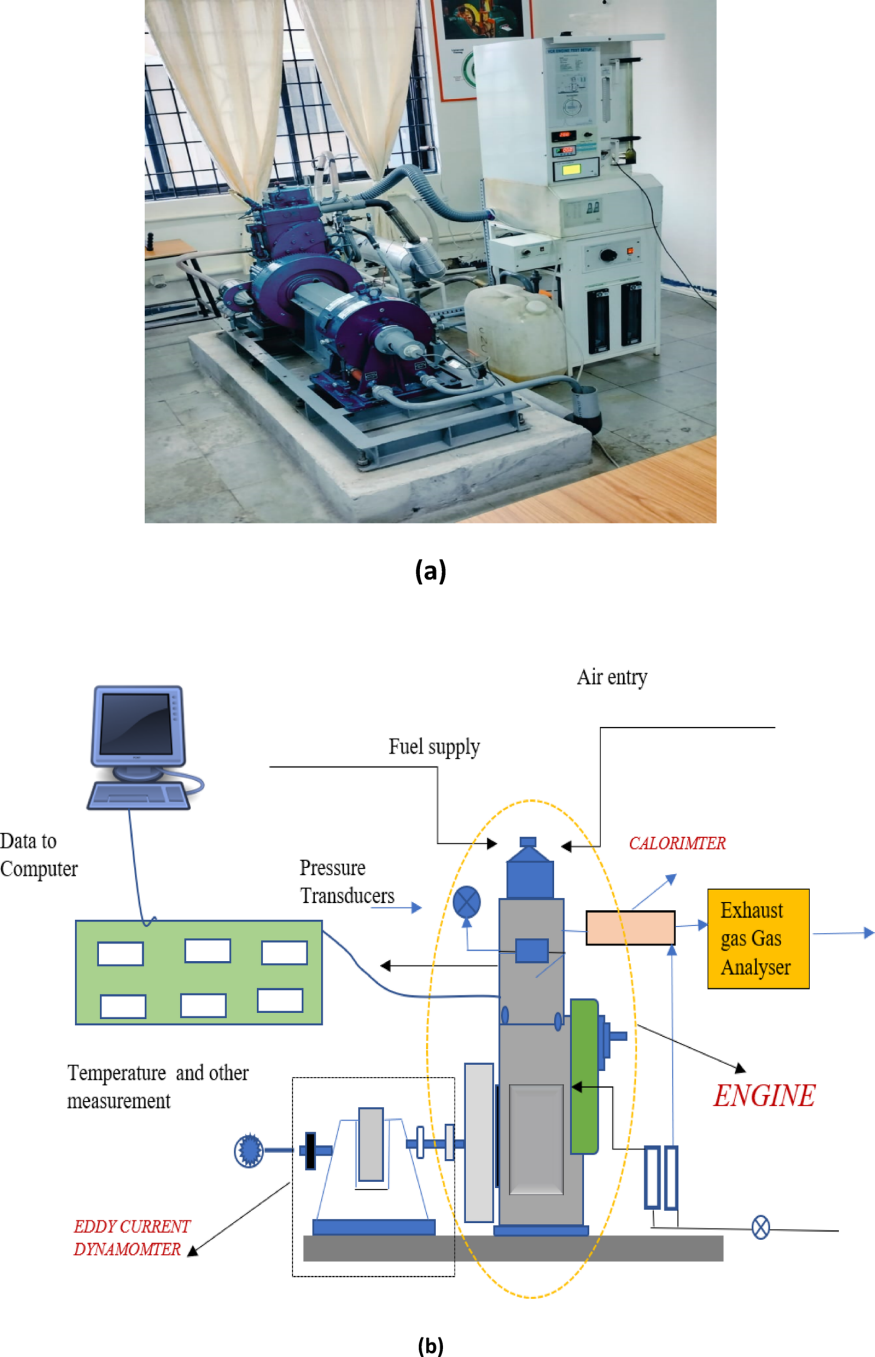
(**a**) Experimental setup (**b**) Schematic of engine setup.

A computerized test setup (Fig. 6a, as detailed in Table 3) with a variable compression ratio diesel engine was employed to record measurements while maintaining a constant engine speed and adjusting the applied load (Fig. 6a). The injection pressure (200 bar), injection timing (27degree bTDC), and compression ratio (17.5:1). The experiments were conducted for 3 trials and average data were taken for interpretation.

The AVL DIGAS 444 N gas analyzer is part of the experimental setup and is a multipurpose gas analyzer used for emission assessment. It quantifies crucial exhaust components including CO, CO_2_, HC, O_2_ and NO_x_. The gas analyzer details, like range, accuracy, and uncertainty, are given in Table 4. The complete engine setup, including accessories, is shown in Fig. 6b.

**Table 3:** Specifications of computerized diesel engine test Rig.

Specification	Details
Engine type	1 Cylinder, 4 Stroke, Constant Speed, Water Cooled, Diesel Engine
Power	3.5 kW @ 1500 rpm
Compression ratio (CR)	17.5:1
Cylinder bore	87.50 mm
Stroke length	110.00 mm
Connecting rod length	234.00 mm
Swept volume	661.45 cc
Orifice diameter	20.00 mm
Orifice coefficient of discharge	0.6
Dynamometer type	Eddy Current
Dynamometer arm length	185 mm

**Table 4:** Specification of engine exhaust gas Analyzer.

Make and model	AVL DIGAS 444 *N*		
Exhaust gas species	Range	Accuracy	Uncertainty
CO (% vol)	0–15	− 0.01 to + 0.01	− 0.01 to + 0.01
HC (ppm)	0 to 2 × 10^4^	− 10 to + 10	− 0.05 to + 0.05
NO_x_ (ppm)	0 to 5 × 10^3^	− 50 to + 50	− 0.01 to + 0.01

### Data preparation and random forest regressor model implementation

**Fig. 7:**
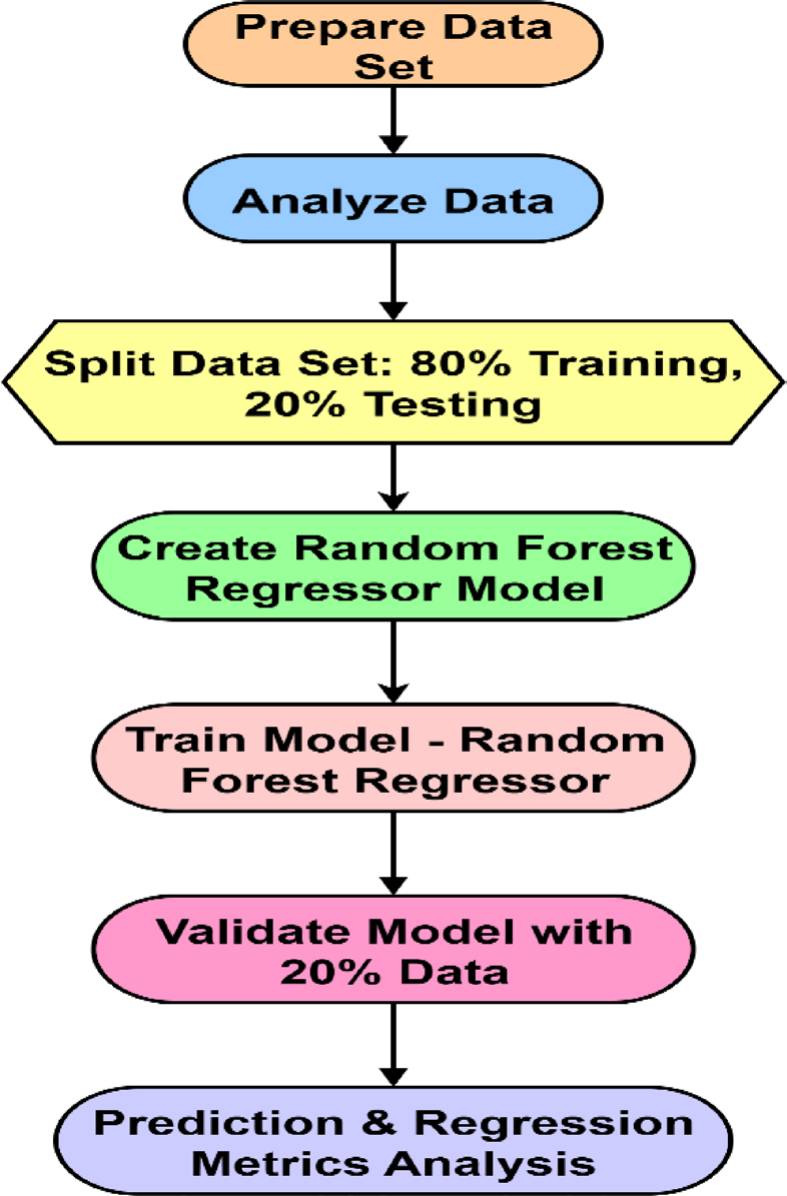
Data preparation and random forest regressor model implementation.

The methodology described herein outlines the process for implementing a Random Forest Regressor (RFR) Model (Fig. 7). Initially, the dataset is prepared and analyzed to ascertain its suitability for modeling. These data is then partitioned into training and testing subsets, with an 80 − 20% division. Subsequently, an RFR Model is constructed and trained using the 80% training data. The model’s performance is validated employing the remaining 20% of the data, and the procedure concludes with an analysis of prediction and regression metrics to evaluate the model’s accuracy and effectiveness.

This research utilized an ML model to predict performance, combustion, and emission characteristics. An RFR Model was trained using a dataset comprising 25 readings of B20UTOME blends with NPs. The model was developed using the Scikit-learn ML library, along with advanced Python libraries such as NumPy, Pandas, and Matplotlib. The input parameters encompassed engine load, biodiesel blend ratio, NPs concentration, oxygen storage capacity, fuel density, fuel viscosity, cetane number, calorific value, and flash point. The output variables included brake thermal efficiency, specific fuel consumption, cylinder pressure, net heat release rate, and emission characteristics (CO, HC, NO_x_, and CO_2_). This comprehensive dataset, incorporating both fuel properties and engine operating conditions, is well-suited for supervised ML. The RFR model was selected for its resilience and capacity to manage nonlinear relationships, mitigating the risk of overfitting.

## Results and discussion

### Characterization of diesel and its blend

**Fig. 8:**
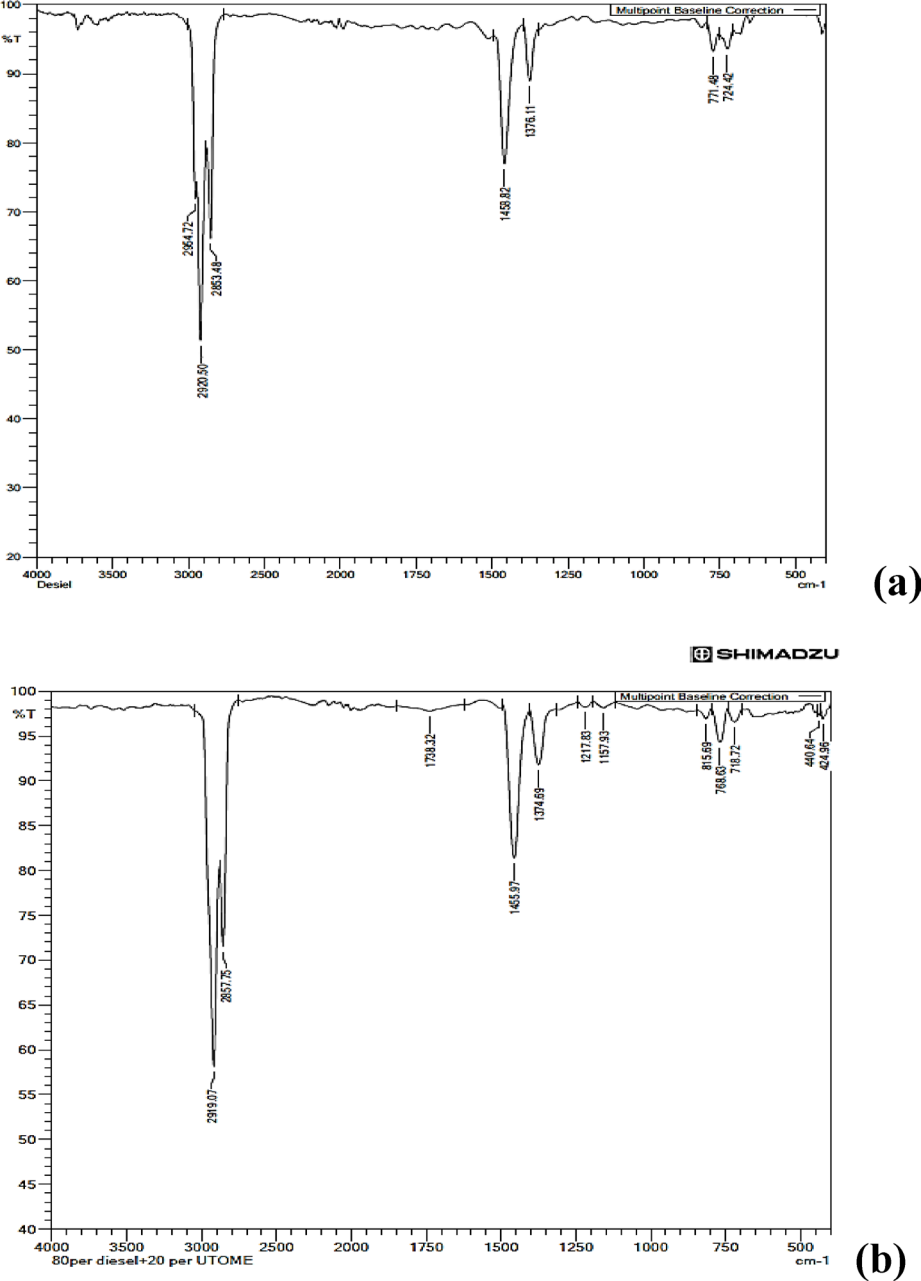
(**a**) FTIR of diesel (**b**) FTIR of B20.

The most common characterization techniques are UV spectroscopy, FTIR, and NMR. The biodiesels used in internal combustion engines are frequently tested for degradation, mixing, and deposit formation, using an infrared (IR) spectroscope (Shimadzu FTIR-8400 S Spectrometer). The range of the Shimadzu spectrophotometer is 4000 –400 cm^− 1^. As an appealing stimulus, spectrum graphs are displayed at a resolution of 4 cm^− 1^. Each spectrum was captured in the 4000 cm-1 to 400 cm-1 range. The FTIR of diesel and B20 fuel are displayed in Fig. 8a and b. These figures illustrate the potential of UTO and UTOME in terms of transmittance, wavelength, and absorbance. Functional groups and the bands corresponding to different stretching and bending vibrations in the biodiesel sample were identified using the infrared FTIR spectra.

Figure 8a shows the FTIR analysis of pure diesel. For pure diesel, strong absorption bands are found in the range 2990–2850 cm^− 1^, which clearly indicates the presence of alkanes having C-H stretch. Figure 8b indicates the FTIR of B20 fuel; the observed peaks are like those of diesel with slight shifts, but some additional peaks with lesser intensities are observed. This indicates there no chemical reactions occurred after mixing. The peak at 2926.13 cm^− 1^(3200−2500 range) indicates the presence of the O-H stretch, corresponding to carboxylic acids. The peak at 722.93 cm^− 1^ corresponds to Alkenes with a C-H bend of aromatic compounds(https://bpb-us-e1.wpmucdn.com/sites.ucsc.edu/dist/9/291/files/2015/11/10-IR-Tables-1ld1zfv.pdf)

### Performance characteristics

#### Brake thermal efficiency (BTE)

**Fig. 9:**
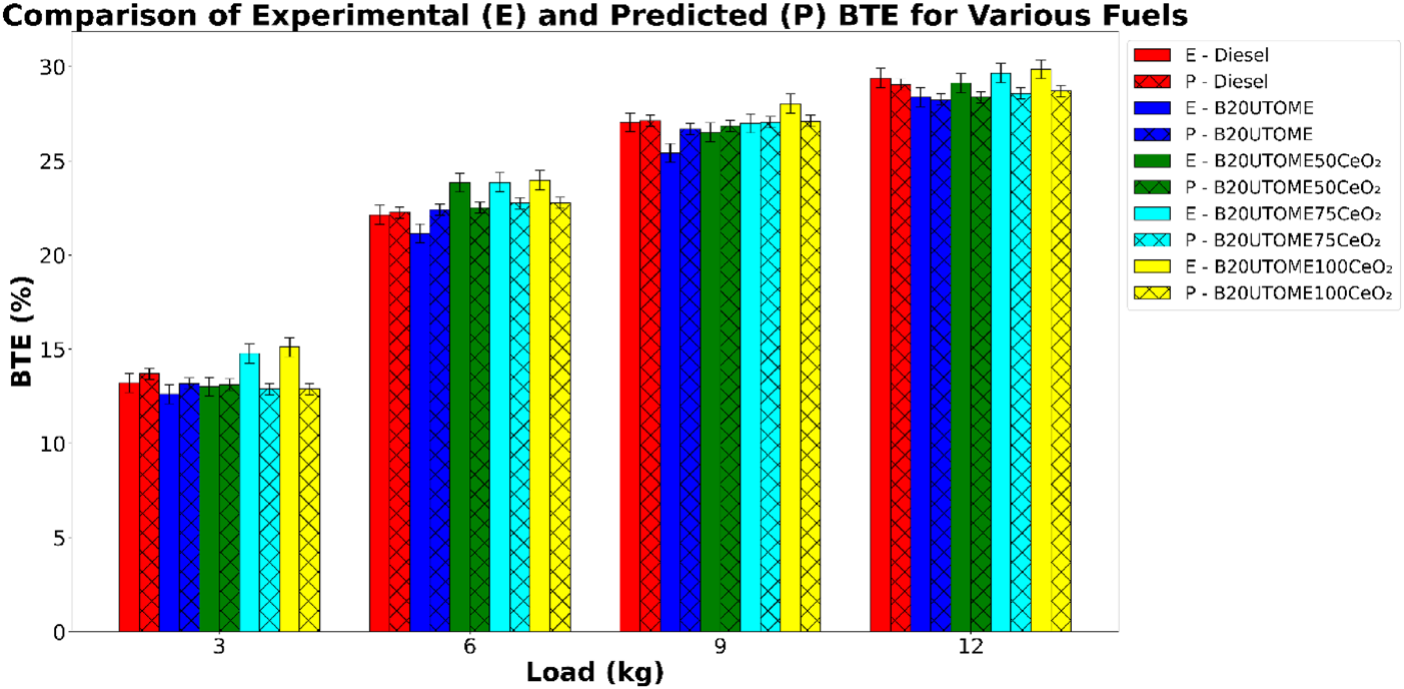
Variation of BTE with various loads and at constant speed.

Figure 9 depicts the variation in BTE at various loads with different fuels and it shows that the BTE increases with increase in load value. This is due to more fuel is burned with increase in load which causes a larger fraction of the released heat is converted into useful work while the relative losses through cooling, exhaust, and friction decrease. At higher loads, the in-cylinder temperature and pressure rise, leading to more complete combustion and reduced unburned fuel losses. It also reveals that adding CeO_2_ NPs to the B20UTOME blend improves BTE, matching the performance of pure diesel. The high surface area-to-volume ratio of these nano additives enhances combustion by facilitating better fuel atomization and quicker evaporation, leading to more efficient energy conversion. The nano-fuel spray exhibited a greater penetration distance, a narrower cone angle, and an increased fuel and air mixing. The raising the concentration of NPs expanded the difference in spray characteristics between nano-fuel and diesel^38^. The CeO_2_ acts as an effective oxygen buffer in combustion systems due to its excellent oxygen storage and release capability. In fuel-rich regions, where oxygen availability is limited, CeO_2_ provides lattice oxygen to promote oxidation of incomplete combustion products such as CO, HC, and soot precursors. This redox property of CeO_2_ accelerates the breakdown of larger HC chains and supports the conversion of elemental carbon into CO_2_. The NPs dosage of 50 ppm results in lower BTE as compared to other dosages. However, a slight variation in BTE was observed with dosages of 75 and 100 ppm of CeO_2_ NPs at full load. The dosage level of 100 ppm results in better combustion and hence the BTE value is higher. Also, the high thermal conductivity and surface activity of CeO_2_ NPs enhance the heat transfer rate in the fuel droplets, improving atomization and vaporization of the biodiesel blend, which normally has high viscosity and poor volatility. The comparison of the predicted and experimental BTE values, as shown in Fig. 9, indicates a close alignment between the two, validating the model’s reliability. The BTE at maximum load for E-B20UTOME100CeO_2_ is 29.85%, while P-B20UTOME100CeO_2_ exhibits a BTE of 28.7%. This 3.85% variation may stem from the random forest model’s inability to fully account for the nonlinear effects of nano-additive interaction and combustion dynamics under high-load conditions. The RFR model used for this validation achieved a high R^2^ score (0.9701) and a low MSE (Mean Squared Error) (2.8911), confirming its accuracy in estimating BTE. These findings highlight the effectiveness of the B20UTOME100CeO_2_ blend as a superior alternative for improving combustion efficiency compared to both B20UTOME without additives and pure diesel.

#### Regression analysis of BTE

**Fig. 10:**
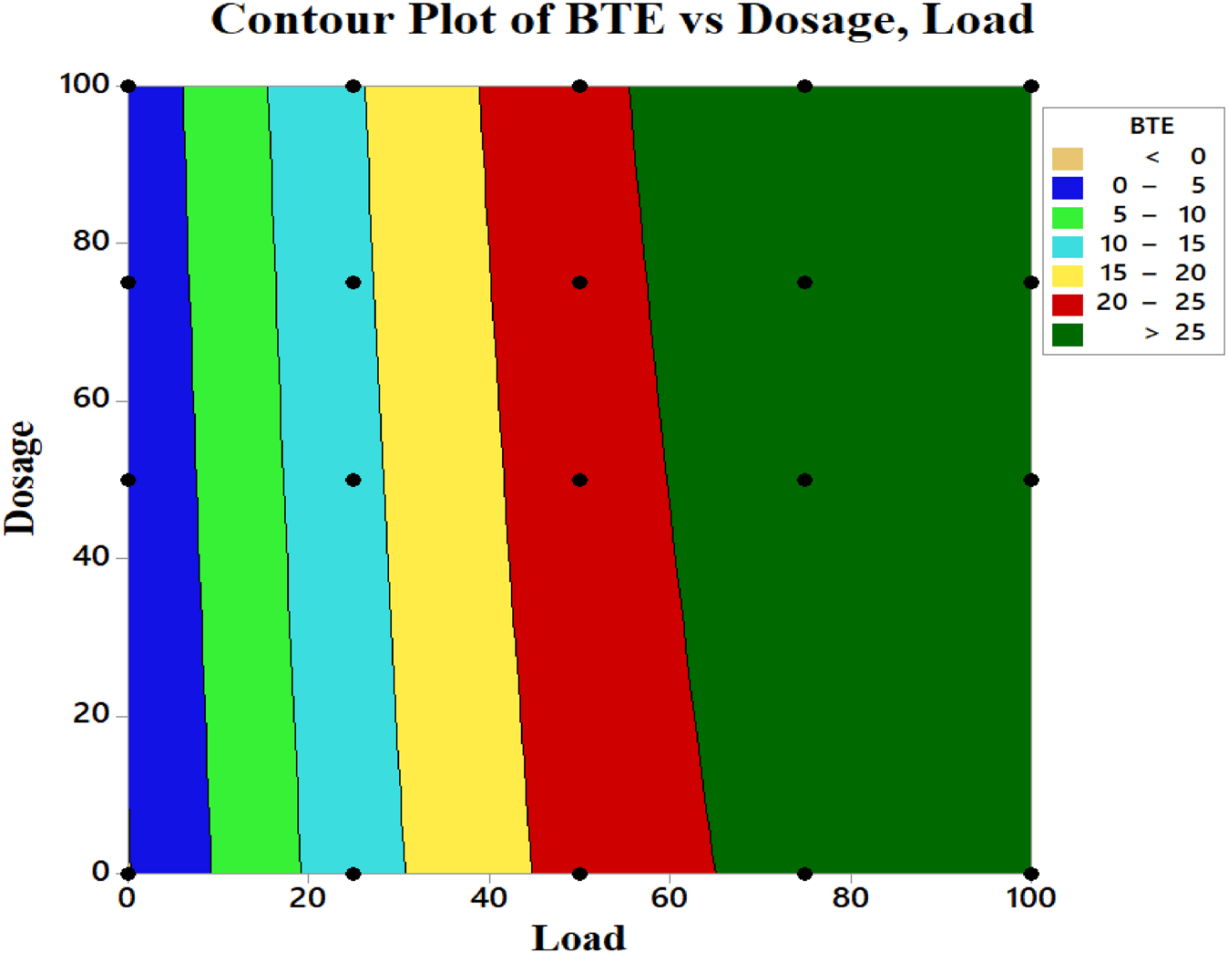
Variation of BTE with respect to various loads and NP dosages.

The interactions between load and dosage were evaluated using statistical RSM analysis. The results obtained, which include the main and interaction effects, model coefficients, and the calculated standard deviation for all coefficients, are summarized in Table 5. Table 6 presents the coded coefficients. From the tables, it can be inferred that the most impactful variables are the concentration of CeO_2_ NPs and the load. The dosage has a positive impact on BTE, as shown in Fig. 10. The variation in BTE was significantly larger for higher loads, while the influence of dosage was found to be substantial. Conversely, as the load diminishes, the variation in BTE also reduces, leading to a limited effect of dosage in that context.

**Fig. 11:**
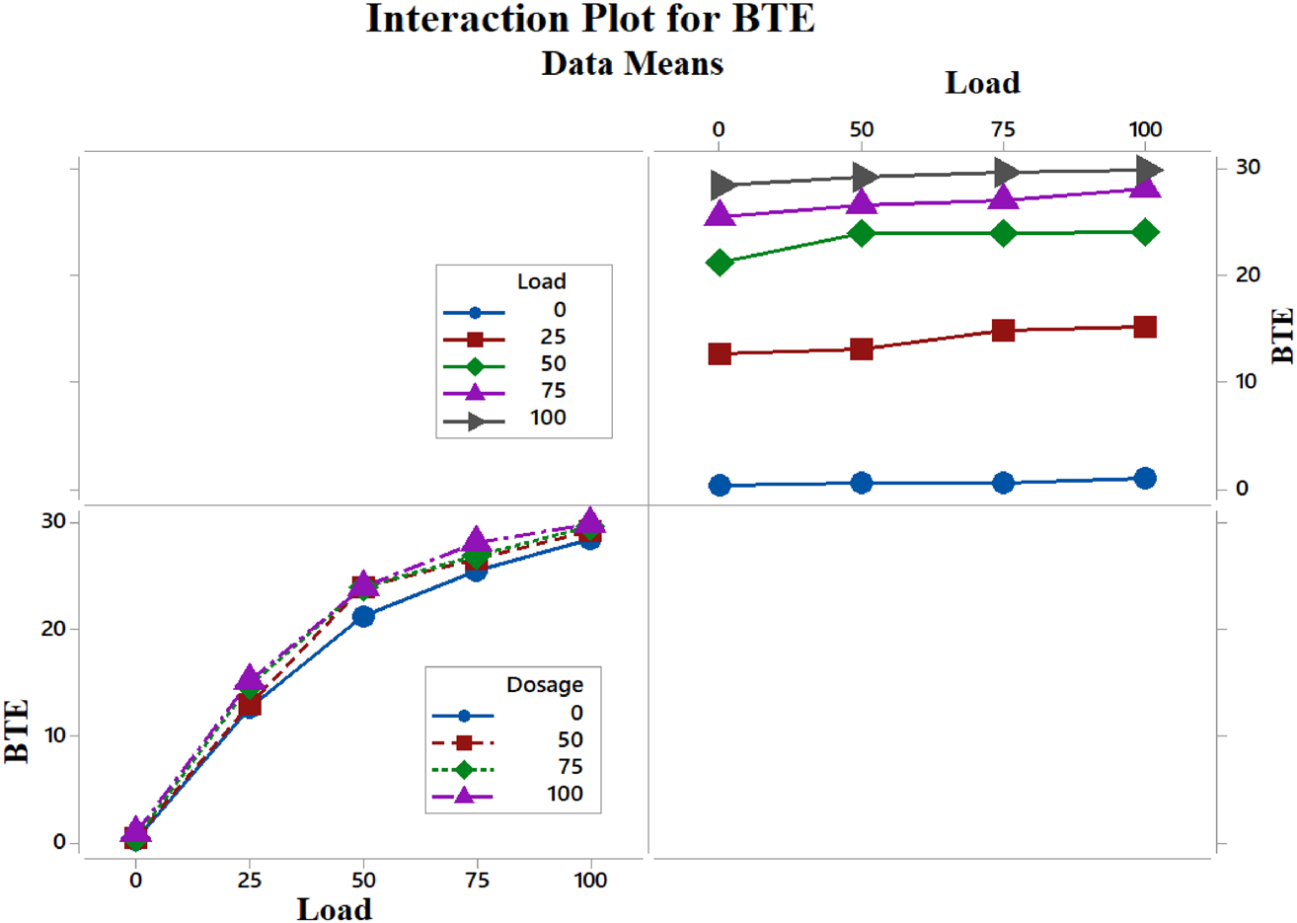
Interaction plots among parameters.

Additionally, a detailed examination of Fig. 10 indicates that BTE values for dosages of 0–50 and 60–100 mg/l, under loads of 75–100% show minimal differences in the former case. This leads to the inference that at concentrations up to 50 mg/l, NPs have a limited impact on BTE, although there is a noticeable upward trend as the concentration approaches 60 mg/l. In contrast, BTE values in the latter case were higher than those in the former; however, there was not much fluctuation beyond 65 mg/l. This suggests that after reaching 65 mg/l, further increases in dosage do not lead to significant changes in BTE. This phenomenon can be attributed to increased CeO_2_ levels, causing atomization issues. The higher concentrations of CeO_2_ may result in agglomeration, which leads to inconsistent fuel quality and subsequently affects BTE due to the combustion of the biodiesel blend. The ideal dosage ranges are found above 65 mg/l, for loads of 80–90%, as illustrated in the higher areas of the dark green bands in the plots. The interaction diagrams demonstrate that there are no interactions between the parametric changes (Fig. 11). The generalized equation for BTE is

$$\begin{aligned} {\text{BTE }} & = -{\text{0}}{\text{.183 + 0}}{\text{.5914 Load + 0}}{\text{.0184 Dosage }} -{\text{ 0}}{\text{.003140 Load}}*{\text{Load}} \\ & \quad -{\text{0}}{\text{.000016 Dosage}}*{\text{Dosage + 0}}{\text{.000066 Load}}*{\text{Dosage}} \\ \end{aligned}$$

### Response surface regression: BTE versus load, dosage

**Table 5:** Analysis of Variance obtained from Response Surface Regression.

Source	DF	Adj SS	Adj MS	F-Value	*P*-Value
Model	5	2202.07	440.41	608.2	0
Linear	2	1925.54	962.77	1329.56	0
Load	1	1914.67	1914.67	2644.11	0
Dosage	1	10.87	10.87	15.01	0.002
Square	2	215.68	107.84	148.92	0
Load*Load	1	215.67	215.67	297.84	0
Dosage*Dosage	1	0.01	0.01	0.01	0.927
2-Way Interaction	1	0.15	0.15	0.2	0.658
Load*Dosage	1	0.15	0.15	0.2	0.658
Error	14	10.14	0.72		
Total	19	2212.21			

Model Summary: S: 0.850956, R-sq: 99.54%, R-sq(adj): 99.38%, R-sq(pred): 99.02%.

.

**Table 6:** Coded coefficients.

Term	Effect	Coef	SE Coef	T-Value	*P*-Value	VIF
Constant		22.579	0.386	58.47	0	
Load	28.067	14.033	0.273	51.42	0	1.03
Dosage	2.008	1.004	0.259	3.87	0.002	1.01
Load*Load	− 15.7	− 7.85	0.455	− 17.26	0	1

#### Specific fuel consumption (SFC)

**Fig. 12:**
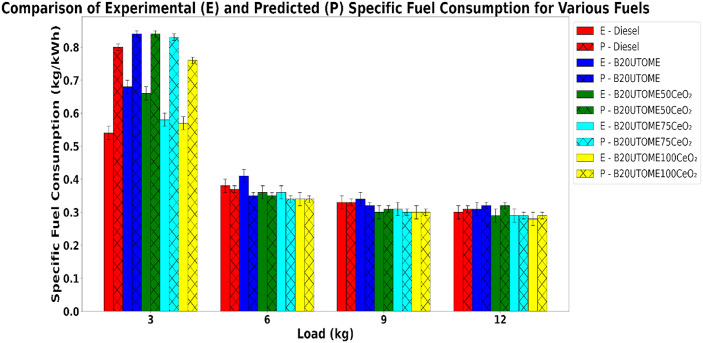
Variation of SFC with various loads and at constant speed.

Figure 12 represents the variation of SFC with various loads and with various fuels and it shows that the SFC of the engine decreases with increasing load because the engine uses fuel more efficiently when producing higher power. At low loads, a significant portion of the fuel’s energy is wasted in overcoming fixed losses such as friction, cooling, and pumping, while only a small part contributes to useful work. As the load increases, these losses remain nearly constant, but the useful power output rises sharply, so the amount of fuel required per unit of power (SFC) drops. Additionally, higher cylinder temperatures and pressures at greater loads promote more complete combustion, improving thermal efficiency and further reducing SFC. The addition of CeO_2_ NPs to the B20UTOME blend resulted in a decrease in Specific Fuel Consumption when compared to both pure diesel and the B20UTOME blend without additives. This enhancement in fuel efficiency can be attributed to improved combustion processes, as the NPs facilitate superior fuel–air mixing and accelerate energy release. Also, the CeO_2_ acts as a combustion promoter by lowering activation energy and increasing the rate of oxidation reactions. The faster and more efficient combustion ensures that energy release occurs at the optimum crank angle, improving thermal efficiency and lowering SFC. Figure 12, which illustrates a comparison between experimental and predicted SFC values, reveals a close correlation, thereby substantiating the predictive model’s accuracy. Specifically, at peak load conditions, the experimental SFC for E-B20UTOME100CeO_2_ was measured at 0.28 kg/kWh, whereas the P-B20UTOME100CeO_2_ model predicted an SFC of 0.29 kg/kWh, representing a discrepancy of approximately 3.57%. This marginal difference suggests that the random forest model may exhibit a slight underestimation of fuel efficiency under high-load operational regimes. In aggregate, the predictive model demonstrates a high degree of precision in forecasting SFC, evidenced by minimal error metrics and a robust correlation with empirical data.

### Combustion characteristics

#### Cylinder pressure (CP)

**Fig. 13:**
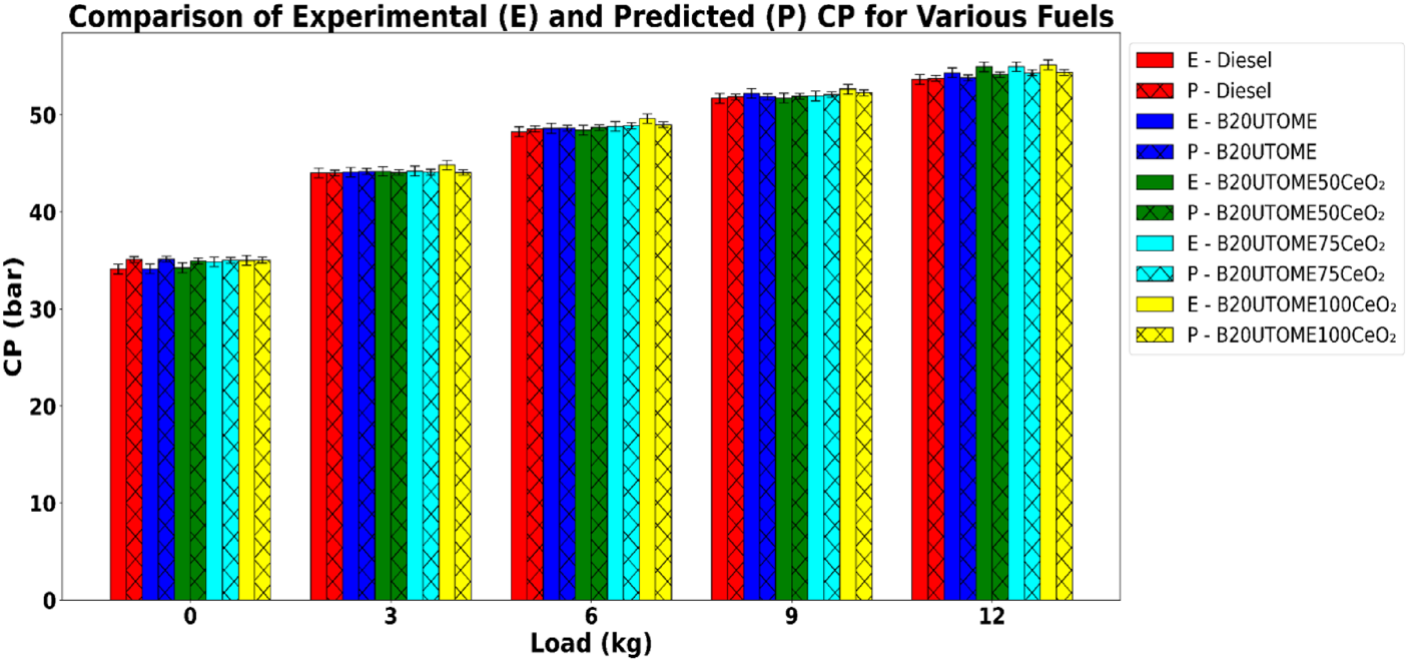
Variation of CP with various load at constant speed.

The variation of CP with varying load at a constant speed is depicted in Fig. 13. The data show that all fuel blends exhibit increased cylinder pressure as the load is increased. This is because more fuel is injected into the cylinder to produce higher power output, leading to greater heat release during combustion. This additional fuel raises the in-cylinder temperature and energy density, causing the air–fuel mixture to expand more vigorously and generate higher peak pressures. At higher loads, combustion also occurs under more favorable conditions such as higher temperature, improved mixing, and faster reaction rates, which further intensify pressure rise. In essence, as load increases, the greater quantity of fuel burned releases more energy per cycle, resulting in higher cylinder pressure and stronger combustion forces that produce greater mechanical work. This figure also shows that the B20UTOME blends demonstrate slightly higher CP compared to pure diesel, which exhibits a consistent rise in CP. Furthermore, the peak cylinder pressure of B20UTOME is further augmented by the addition of CeO_2_ NPs, with B20UTOME100CeO_2_ achieving the maximum cylinder pressure across all load conditions. The optimized CeO_2_ NPs dosage of 100 ppm, which promotes improved fuel atomization and dispersion, is responsible for the reduction in ignition delay. The lower ignition delay and increase in flame speed cause the main combustion phase to occur closer to top dead center. This optimized combustion phasing maximizes pressure rise within the cylinder. Under maximum load conditions, E-B20UTOME100CeO_2_ yields a cylinder pressure of 55.15 bar, whereas P-B20UTOME100CeO_2_ results in 54.37 bar. This represents a variation of about 1.41%, potentially attributable to minor inconsistencies in the random forest model’s peak pressure predictions, particularly when influenced by intricate combustion dynamics at full engine load. The accuracy of the predicted cylinder pressure values was validated using a RFR model, which demonstrated a low MSE (0.3061) and a high R^2^ score (0.9939), indicating a close correspondence with the experimental results.

#### Net heat release (NHR)

**Fig. 14:**
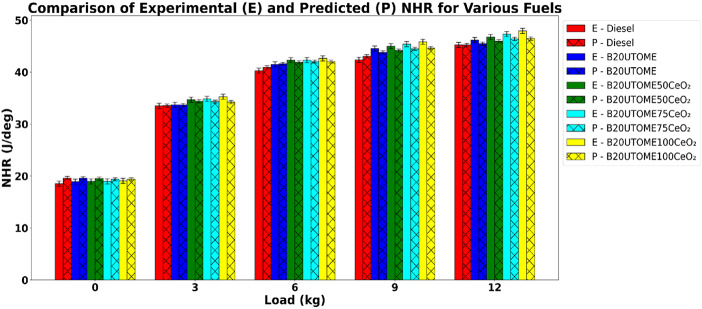
Variation of NHR with various loads and at constant speed.

The heat release rate was determined using the following equation.

$$\:\frac{dQ}{d\theta}=\frac{\gamma}{\gamma-1}p\frac{dV}{d\theta}+\frac{1}{\gamma-1}\frac{Vdp}{d\theta}$$

γ-specific heat ratio, p-cylinder pressure, *V*-cylinder volume, $\:\frac{dV}{d\theta}$-rate of change of cylinder volume with crank angle, $\:\frac{dp}{d{\theta}}$-rate of change of cylinder pressure with crank angle.

The study examined the net heat release (NHR) values of diesel and comparable fuel blends. Figure 14 depicts that the NHR increases with an increase in load, and this is because higher loads require more fuel to be injected and burned per cycle. With more fuel, a greater amount of chemical energy is released during combustion, producing a faster and larger energy release over the crank-angle. At higher loads, the in-cylinder pressure and temperature are also higher, which accelerates the combustion reactions and reduces ignition delay, leading to a sharper and more intense heat release. Also improved air–fuel mixing and more complete combustion at higher loads contribute to a higher NHR, reflecting the increased energy conversion per cycle. The incorporation of CeO_2_ NPs additives into the B20UTOME blend further enhanced the NHR, and this can be attributed to the enhanced catalytic activity of the NPs, which promotes improved fuel atomization, reduced ignition delay, and a more complete and efficient combustion process. Among all the dosages considered, the 100 ppm dosage with B20UTMO (B20UTOME100CeO_2_) mixture exhibited the highest NHR across all tested load conditions, indicating the most substantial improvement. This is due to optimized CeO_2_ NPs dosage, which promotes oxidation of intermediate species and soot precursors, minimizing incomplete combustion. The cleaner and faster burning process increases the total energy released per cycle and improves combustion stability. The comparison of predicted and experimental NHR values, as shown in Fig. 14. At maximum load, E-B20UTOME100CeO_2_ exhibits a normalized heat release of 47.92 J/deg, while P-B20UTOME100CeO_2_ demonstrates a value of 46.46 J/deg. This represents a percentage variation of approximately 3.04%, potentially stemming from the model’s inherent limitations in accurately representing the transient heat release behavior induced by nano-additives during combustion under elevated load conditions. Figure 14 indicates a close alignment, demonstrating the high predictive accuracy and reliability of the RFR Model used for validation, which achieved an MSE of 0.8167 and a high R^2^ score of 0.9917.

### Emission characteristics

#### Carbon monoxide (CO)

**Fig. 15:**
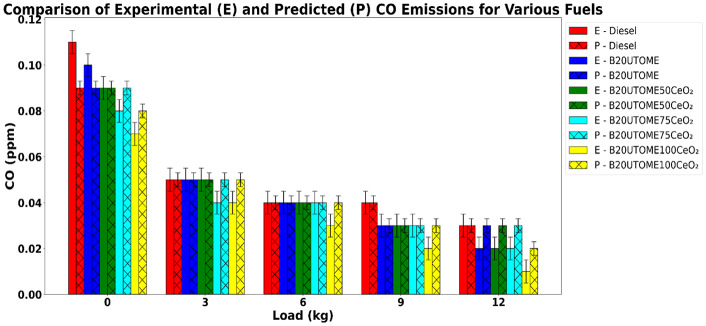
Variation of CO with various loads and at constant speed.

Figure 15 depicts the relationship between engine load and carbon monoxide emissions at a constant speed. For all fuel types, CO emission decreases, and this is because higher loads improve combustion conditions. At higher loads, more fuel is injected, raising the cylinder temperature and pressure, which promotes more complete oxidation of carbon in the fuel to CO_2_ rather than CO. The shorter ignition delay and faster combustion under these conditions reduce the formation of partially oxidized species like CO. Also, the higher in-cylinder oxygen availability and better mixing at increased loads help ensure that carbon atoms are fully converted, resulting in lower CO emissions. Compared to pure diesel, B20UTOME blends exhibit lower CO emissions, with B20UTOME100CeO_2_ demonstrating the lowest emissions across all loads. This reduction is attributed to the oxygen content in CeO_2_ NPs, which promotes more thorough air-fuel mixing, enhances combustion efficiency, and shortens the ignition delay period. The shorter ignition delay promotes a faster combustion rate, leading to higher in-cylinder temperatures that favor the oxidation of CO into CO_2_. This thermal and kinetic advantage helps minimize CO emissions. E-B20UTOME100CeO_2_ emits 0.01 ppm CO at maximum load, whereas P-B20UTOME100CeO_2_ emits 0.02 ppm. The 50% variation is attributable to the minimal absolute CO values at full load. Such sensitivity to minor absolute differences at these low emission levels results in a substantial percentage variation, highlighting the model’s predictive sensitivity to changes in CO emissions. The RFR model, characterized by a low Mean Squared Error (0.0001382) and an R-squared score of 0.8237, accurately predicted the CO levels, indicating reliable predictive performance and highlighting the role of CeO_2_ NPs in achieving cleaner combustion.

#### Hydrocarbons (HC)

**Fig. 16:**
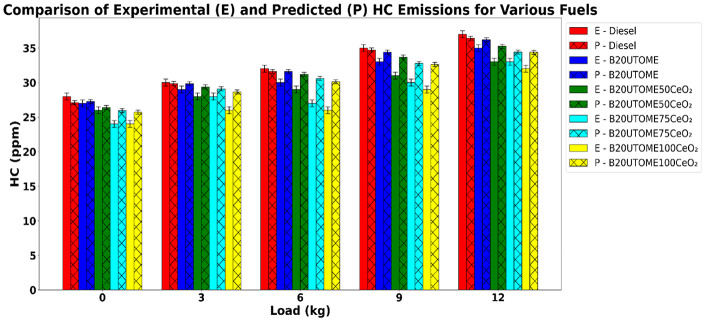
Variation of HC with various loads and at constant speed.

The hydrocarbon emissions fluctuate with varying load levels at a constant speed, as depicted in Fig. 16. It shows that HC value increases with increase in load as higher loads require larger fuel injections, which can lead to incomplete combustion in certain regions of the cylinder. At high loads, the cylinder temperature and pressure rise overall; however, some localized zones, such as near the cylinder walls or in fuel-rich pockets, may not reach a sufficient temperature for complete oxidation. The larger fuel quantity also increases spray penetration and fuel-rich zones, where oxygen is limited, resulting in unburnt HC escaping into the exhaust. Also, shorter residence time of fuel in these regions at high load can prevent complete combustion, causing HC emissions to rise despite the overall higher combustion efficiency. While B20UTOME blends exhibit slightly lower emissions compared to pure diesel across all load levels, HC emissions increase with rising load for all fuel types. The addition of CeO_2_ NPs to B20UTOME significantly reduces HC emissions, with the decreases becoming more pronounced at higher CeO_2_ concentrations. By lowering the activation energy and enhancing flame propagation speed, B20UTOME100CeO_2_ ensures that a greater portion of the injected fuel undergoes complete combustion within the cylinder, leaving fewer HC in the exhaust across all load conditions. At maximum load, E-B20UTOME100CeO_2_ produces 32 ppm of HC emissions, while P-B20UTOME100CeO_2_ produces 34.38 ppm. The approximately 7.44% difference may stem from the model’s tendency to slightly overpredict unburnt hydrocarbon levels, potentially due to nonlinear effects of combustion characteristics and additive dispersion at higher loads. The RFR Model has an MSE of 0.7160 and an R^2^ score of 0.9295, validatinga MSE of 0.7160 and an R^2^ score of 0.9295 validates the predicted HC emissions, demonstrating excellent predictive accuracy and emphasizing the catalytic role of CeO_2_ NPs in reducing emissions.

#### NO_x_ (Nitrogen Oxides)

**Fig. 17:**
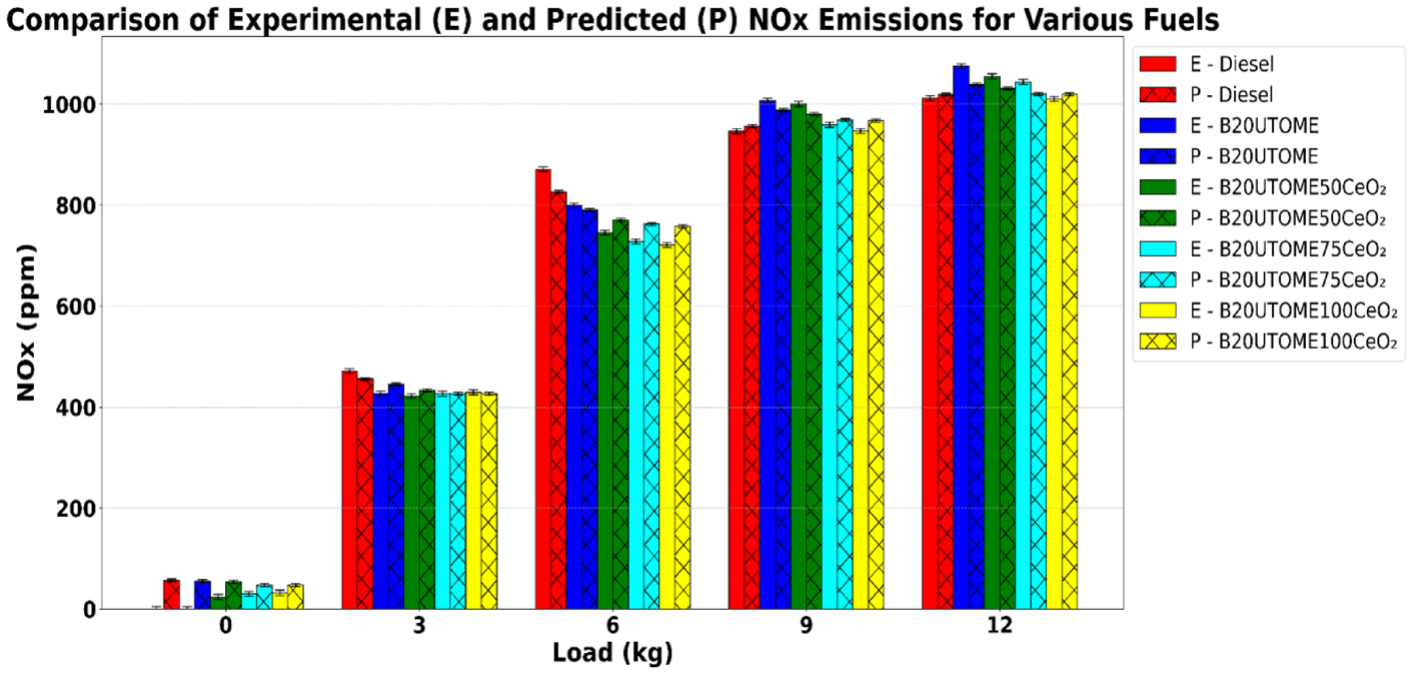
Variation of NO_x_ with various loads and at constant speed.

The fluctuation of NO_x_ emissions as a function of load at constant engine speed is depicted in Fig. 17. For all fuel blends, NO_x_ emissions increase with higher engine loads as the higher loads lead to higher in-cylinder temperatures and pressures, which strongly favor the thermal formation of nitrogen oxides. As load increases, more fuel is injected, raising the peak combustion temperature; since NOₓ formation is highly temperature dependent, even small increases in peak temperature result in exponentially higher NOₓ production. Also, the higher oxygen availability and faster combustion at elevated loads provide the conditions necessary for nitrogen in the air to react with oxygen, further increasing NOₓ emissions. Thus, the combination of higher temperature, pressure, and oxygen-rich environment at increased load directly leads to higher NOₓ formation. The pure diesel fuel exhibits a steady rise in NO_x_ emissions, whereas B20UTOME blends generate higher NO_x_ levels at greater loads owing to the oxygen content of biodiesel, which enhances combustion. Incorporating CeO_2_ NPs onto B20UTOME results in substantial reductions in NO_x_ emissions, with higher CeO_2_ NPs concentrations leading to more significant decreases. The CeO_2_ improves combustion efficiency and thermal management, lowering peak temperatures and consequently reducing NO_x_ formation. The B20UTOME100CeO_2_ blend demonstrates the lowest NO_x_ emissions among all the tested blends. At the maximum load, NO_x_ emissions are 1010 ppm for E-B20UTOME100CeO_2_ and 1019.28 ppm for P-B20UTOME100CeO_2_. This represents a 0.92% variation, indicative of acceptable model prediction accuracy. The slight deviation may arise from subtle differences in the model’s simulation of temperature-dependent NO_x_ formation mechanisms during combustion under high engine load conditions. The predicted values closely align with the experimental data, validated by the RFR Model with an R-squared score of 0.9836, confirming the model’s strong predictive accuracy.

The temple oil does not have any commercial value in India; however, if it is used as a feedstock, the cost of producing biodiesel in India will be significantly reduced. Consider the cost of temple oil collection, filtration, and storage as 0.11$ per liter at the collection center. In a typical transesterification process, the major expenses apart from feedstock are methanol, catalyst (both acid and base catalysts), and processing costs, including electricity, water, labor, and maintenance. With a conversion efficiency of approximately 90%, the effective raw biodiesel production cost per liter of oil is close to $0.54. After adding water wash, removing moisture, and purifying crude biodiesel, and accounting for overhead expenses, the cost of fuel is the cost of temple oil collection, filtration, and storage, which is $0.11 per liter at the collection center. Grade biodiesel is roughly 0.67$ per liter^39^.

In comparison, retail diesel prices in India (as of September 2025) are around ₹ 95 (approximately $ 1.08 per liter), depending on the city. Thus, biodiesel from temple oil at this feedstock price could be produced at nearly two-thirds of the cost of petroleum diesel, offering a large economic advantage. Therefore, with efficient temple oil collection and large-scale processing, biodiesel production at a feedstock cost of ₹10/L (0.11 $) is not only technically feasible but also economically very attractive in India.

## Conclusion

This study investigates the feasibility of using B20 biodiesel blend derived from non-edible and underutilized UTO, enhanced with CeO_2_ NPs, as a sustainable alternative to conventional diesel fuel. The RFR model was employed to accurately predict key performance metrics, including BTE, CP, NHR, and emission characteristics. The B20UTOME100CeO_2_ blend demonstrated improved performance characteristics under maximum load conditions and the brake thermal efficiency increased by 1.57% compared to pure diesel. Furthermore, the blend achieved a 2.83% increase in cylinder pressure and a 5.95% improvement in net heat release relative to diesel fuel. The emission analysis showed that carbon monoxide emissions were significantly reduced by approximately 66.67%, and hydrocarbon emission was reduced by 13.51% compared to diesel. Moreover, nitrogen oxides emissions from B20UTOME100CeO_2_ were 6.04% lower than B20UTOME and slightly lower (0.20%) than diesel, attributable to enhanced combustion. The findings indicate that the B20UTOME100CeO_2_ blend exhibited significant improvements in thermal efficiency and combustion, along with reductions in hydrocarbons, carbon monoxide, and nitrogen oxides, although a slight increase in carbon dioxide emissions was observed due to enhanced combustion. The robustness of the model’s predictions was validated through high R^2^ scores and low mean squared error. These results establish B20UTOME with CeO_2_ additives as a viable alternative to traditional diesel, offering enhanced performance, cleaner combustion, and reduced environmental impact.

### Limitations of the present study

Work was carried out for limited number loads, limited concentrations of CeO_2_ NPs.

### Recommendations for future work

- A computational fluid dynamics (CFD) study can be carried out to investigate the effect of CeO_2_ on fuel atomization and spray formation.
- The effect of process variables such as compression ratio, injection timing, on the engine’s emissions and performance can be studied and optimized.
- AI-based predictions can be performed using different AI models and the results can be compared.
- A study can be carried out to explore methodologies for enhanced NO_x_ emission reduction.

## Data Availability

The authors declare that the findings of this study are available within the paper, and the datasets used and analyzed during the current study are available from the corresponding author on reasonable request.

## Abbreviations

AI: Artificial Intelligence
ANN: Artificial Neural Network
APS: Average particle size
ABE: Acetone–Butanol–Ethanol
ANOVA: Analysis of variance
B10: 90% Diesel + 10% biodiesel
B20: 80% Diesel + 20% biodiesel
B20UTOME: 80% Diesel + 20% Used Temple Oil Methyl Ester
B20UTOME50CeO_2_: B20UTOME with 50 ppm CeO_2_ NPs
B20UTOME75CeO_2_: B20UTOME with 75 ppm CeO_2_ NPs
B20UTOME100CeO_2_: B20UTOME with 100 ppm CeO_2_ NPs
B20UTOME100CeO_2_ E: Experimental result value
B20UTOME100CeO_2_ P: Predicted result value
B25: 75% Diesel + 25% biodiesel
B30: 70% Diesel + 30% biodiesel
B40: 60% Diesel + 40% biodiesel
BSFC: Brake specific fuel consumption
BSEC: Brake specific energy consumption
BTE: Brake thermal efficiency
C20Ba100: 80% Diesel + 20% castor biodiesel + 100 ppm BaTiO_3_
CeO_2_: Cerium oxide
CI: Compression ignition
CNT: Carbon nanotube
CNG: Compressed natural gas
CO: Carbon monoxide
CP: Cylinder pressure
CR: Compression ratio
DEE: Diethyl ether
DPA: Diphenylamine
DT: Decision tree
FCC: Face centered cubic
FTIR: Fourier transform infrared spectroscopy
GHG: Greenhouse gas
HC: Hydrocarbon
HRR: Heat release rate
IC: Internal combustion
IMEP: Indicated mean effective pressure
KNN: K nearest neighbors
MAH B20 AL100 CE100: 80% Diesel + 20% Mahua oil + Al_2_O_3_ 100 ppm + CeO_2_ 100 ppm
ML: Machine learning
MSE: Mean squared error
NBD: Neem biodiesel
NHR: Net heat release
NME: Neem methyl ester
NMR: Nuclear magnetic resonance
NO_x_: Nitrogen oxides
NP: Nanoparticle
PAH: Polycyclic aromatic hydrocarbons
RFR: Random forest regression
RSM: Response surface methodology
OSC: Oxygen storage capacity
SVM: Support vector machines
UTO: Used temple oil
UTOME: Used temple oil methyl ester
UV: Ultraviolet
WCO: Waste cooking oil
